# Evaluation of Aptima Zika Virus Assay

**DOI:** 10.1128/JCM.00603-17

**Published:** 2017-06-23

**Authors:** Ping Ren, Daniel A. Ortiz, Ana C. B. Terzian, Tatiana E. Colombo, Mauricio L. Nogueira, Nikos Vasilakis, Michael J. Loeffelholz

**Affiliations:** a Department of Pathology, University of Texas Medical Branch, Galveston, Texas, USA; b Faculdade de Medicina de São José do Rio Preto (FAMERP), São José do Rio Preto, SP, Brazil; c Department of Pathology and Member, Center for Biodefense and Emerging Infectious Diseases, Center for Tropical Diseases, and Institute for Human Infections and Immunity, University of Texas Medical Branch, Galveston, Texas, USA; Memorial Sloan Kettering Cancer Center

**Keywords:** Aptima Zika virus assay, sensitivity, specificity

## Abstract

The Zika virus (ZIKV) epidemic in the Americas poses a public health emergency that requires a swift response. Accurate and reliable ZIKV diagnostic tests serve as an important tool for limiting the spread of ZIKV infections. The Aptima Zika virus assay (Hologic, Marlborough, MA) performed on the automated Panther system is a rapid and high-throughput method for detecting ZIKV RNA using transcription-mediated amplification (TMA) technology. We evaluated the performance characteristics of the Aptima Zika virus assay on clinical serum and urine specimens (*n* = 124) from two different patient populations and samples spiked with ZIKV from three different lineages (*n* = 10). Compared to the real-time reverse transcription-PCR (rRT-PCR) reference method, the Aptima ZIKV assay detected ZIKV RNA with a diagnostic accuracy of 94.8% (95% confidence interval [CI], 89.4 to 97.6), a sensitivity of 94.7% (95% CI, 73.5 to 99.9), and a specificity of 94.8% (95% CI, 88.9 to 97.8). Similar results were obtained regardless of whether a serum or urine source was used. The limits of detection of the assay at a 95% detection probability were 11.5 genome copy equivalents (GCE)/ml (95% fiducial limits, 7.9 to 20.2) in serum and 17.9 GCE/ml (95% fiducial limits, 13.1 to 27.5) in urine. The Aptima Zika virus assay results were highly reproducible (99%), and no cross-reactivity was seen during the testing of a panel of 95 specimens with potentially interfering substances, such as clinically relevant bacteria, fungi, and viruses, including other flaviviruses. The excellent performance characteristics and the convenience of a fully automated testing system make the Aptima ZIKV assay an attractive choice for clinical laboratories detecting ZIKV RNA from serum and urine.

## INTRODUCTION

Zika virus (ZIKV), a member of the Flaviviridae family, is named after the Ziika Forest in Uganda, Africa, from which it was first identified in 1947 (1). The virus is primarily transmitted between humans by *Aedes* spp. mosquitoes, the same vectors that also transmit dengue, yellow fever, and chikungunya viruses. Prior to 2007, only 13 cases of human ZIKV infections were reported, which is partially attributed to the mild nature of ZIKV infection (2). Up to 80% of individuals infected with ZIKV are asymptomatic, while symptomatic individuals display a broad range of symptoms that may include fever, headache, rash, arthralgia, conjunctivitis, and myalgia. However, complications of ZIKV infection have been shown to cause congenital microcephaly and trigger Guillain-Barré syndrome (3). As of February 2017, a total of 76 countries and territories reported cases of ZIKV transmission, with active ZIKV transmission still occurring in the Pacific islands and the Americas (4). The increasing geographical range of ZIKV stresses the importance of diagnostic laboratory tests in confirming infection and mitigating the spread of ZIKV infection.

A diagnosis of ZIKV infection is confirmed by the detection of viral components (e.g., viral RNA, viral proteins, or virus isolation) and/or the host humoral immune response. At early stages of infection (≤14 days after symptom onset), ZIKV RNA can be detected in whole blood (also serum and plasma) and urine samples from patients (5, 6). Real-time reverse transcription-PCR (rRT-PCR) is commonly used for ZIKV RNA detection because of its high sensitivity and specificity. The U.S. Centers for Disease Control and Prevention (CDC) Trioplex real-time RT-PCR assay was the first diagnostic test granted emergency use authorization (EUA) by the Food and Drug Administration (FDA) for the qualitative detection and differentiation of ZIKV RNA from that of dengue and chikungunya viruses (7). Additional EUA-approved ZIKV assays have since been developed, yet their diagnostic utility in a clinical setting has not been thoroughly evaluated.

The Aptima Zika virus assay (Hologic, Marlborough, MA) obtained EUA approval from the FDA on June 17, 2016, for the qualitative detection of ZIKV RNA from human serum, plasma, and urine (collected alongside a patient-matched serum or plasma specimen) (8). The Aptima ZIKV assay uses transcription-mediated amplification (TMA) that targets two highly conserved sequences in the NS2 and NS4/NS5 regions of ZIKV RNA (9). The entire assay is performed in a single tube on an automated Panther system, and initial results are produced in 3.5 h. In this study, we evaluated the performance characteristics of the Aptima Zika virus assay on clinical and spiked specimens at the University of Texas Medical Branch (UTMB) clinical microbiology laboratory.

## RESULTS

### Diagnostic accuracy.

The diagnostic accuracy of the Aptima ZIKV assay was evaluated using clinical serum and urine specimens collected from two different patient populations, along with a subset of ZIKV-spiked samples.

In our service area, 104 clinical specimens from 70 patients met inclusion criteria for ZIKV RNA testing. Female patients comprised 84% (59/70) of the total test volume, and 67% (40/59) of the females were pregnant women. Thirty-eight patients displayed one or more symptoms of ZIKV infection such as headache, fever, rash, conjunctivitis, and joint or muscle pain. Among the 104 U.S. clinical samples tested by the Aptima ZIKV assay, 3 serum and 3 urine specimens tested positive for ZIKV RNA (Table 1). These positive specimens were paired serum and urine specimens from three patients who were symptomatic and had recent travel to an area with active ZIKV transmission (Dominican Republic, Barbados, and Guatemala). Only one pair of serum and urine samples out of these three paired Aptima ZIKV assay-positive samples correlated with rRT-PCR results. The diagnostic accuracy of the Aptima ZIKV assay in testing 104 specimens from our service area was 95.2% (95% confidence interval [CI], 89.0 to 98.2). The diagnostic accuracies were similar regardless of whether the sample was from a serum or urine source.

**TABLE 1:** Aptima ZIKV assay results compared to rRT-PCR results in clinical and spiked specimens

Aptima ZIKV assay result	rRT-PCR results (no.)	
	Positive	Negative
Clinical specimens (United States)		
Serum (*n* = 70)		
Positive	1	2
Negative	1	66
Urine (*n* = 34)		
Positive	1	2
Negative	0	31
Clinical specimens (Brazil)		
Serum (*n* = 20)		
Positive	10	2
Negative	0	8
Spiked specimens		
Serum (*n* = 5)		
Positive	3	0
Negative	0	2
Urine (*n* = 5)		
Positive	3	0
Negative	0	2

The utility of the Aptima ZIKV assay in a patient population from an area in which ZIKV is endemic was assessed using 20 serum samples from Brazilian residents who had previous ZIKV rRT-PCR testing results by a previously described method (5). All patients displayed two or more symptoms of ZIKV infection, and were tested within 6 days of symptom onset by rRT-PCR for ZIKV, dengue virus serotypes 1 to 4 (10), and chikungunya virus (11). Among the 20 samples received, 10 were positive and 10 were negative for ZIKV infection. All patient specimens were negative for dengue virus serotypes 1 to 4 and for chikungunya virus. The diagnostic accuracy of the Aptima ZIKV assay in the Brazilian patient population was 90% (95% CI, 68.7 to 98.4) (Table 1).

The ability of the Aptima ZIKV assay to detect Zika viruses of different lineages was assessed by using a panel of spiked ZIKV-negative specimens. A total of five serum and five urine samples were spiked with one of the following: Mexican ZIKV strain (Mex-81), Cambodian ZIKV strain (FSS13025), African ZIKV strain (DAK), dengue virus, or no virus. The Aptima ZIKV assay and the rRT-PCR reference method accurately identified all samples.

In summary, a total of 134 samples were tested by the Aptima ZIKV assay and the rRT-PCR reference method. Compared to the reference method, the Aptima ZIKV assay displayed a diagnostic accuracy of 94.8% (95% CI, 89.4 to 97.6) with a sensitivity of 94.7% (95% CI, 73.5 to 99.9) and a specificity of 94.8% (95% CI, 88.9 to 97.8) (Table 2).

**TABLE 2:** Overall performance characteristics of the Aptima ZIKV assay (*n* = 134)

Aptima ZIKV assay result	rRT-PCR results		Sensitivity (% [95% CI])	Specificity (% [95% CI])	Diagnostic accuracy (% [95% CI])
	Positive	Negative			
Positive	18	6	94.7 (73.5–99.9)	94.8 (88.9–97.8)	94.8 (89.4–97.6)
Negative	1	109			

### Limits of detection.

The limits of detection (LoD) of the Aptima ZIKV assay were determined by serial dilutions of ZIKV stock (Asian lineage; FSS13025) in ZIKV-negative serum and urine. Final urine dilutions were mixed with urine transport media to simulate clinical collection practices. Several replicates were tested at each dilution to calculate the lowest concentration reliably detected. The LoD of the Aptima ZIKV assay were 11.5 genome copy equivalents (GCE)/ml (95% fiducial limits, 7.9 to 20.2) in serum, and 17.9 GCE/ml (95% fiducial limits, 13.1 to 27.5) in urine at a 95% detection probability (Table 3).

**TABLE 3:** Serial dilutions of ZIKV stock (2.18 × 10^9^ GCE/ml) in serum and urine for limits of detection

Dilution factor	Presumptive GCE/ml	Serum			Urine		
		No. of samples	No. (%) positive	95% CI	No. of samples	No. (%) positive	95% CI
1:100	21,800,000	1	1 (100)	2.5–100	1	1 (100)	2.5–100
1:1,000	2,180,000	1	1 (100)	2.5–100	1	1 (100)	2.5–100
1:10,000	218,000	1	1 (100)	2.5–100	1	1 (100)	2.5–100
1:100,000	21,800	1	1 (100)	2.5–100	1	1 (100)	2.5–100
1:1,000,000	2,180	1	1 (100)	2.5–100	1	1 (100)	2.5–100
1:10,000,000	218	20	20 (100)	83–100	20	20 (100)	83–100
1:100,000,000	21.8	50	50 (100)	93–100	50	49 (98)	89–100
1:200,000,000	10.9	50	46 (92)	81–98	50	42 (84)	71–93
1:1,000,000,000	2.18	50	33 (66)	51–79	50	12 (24)	13–38
1:2,000,000,000	1.09	50	9 (18)	9–31	50	3 (6)	1–17
1:20,000,000,000	0.109	50	1 (2)	0.1–11	50	0 (0)	0–7
1:200,000,000,000	0.0109	30	0 (0)	0–12	30	0 (0)	0–12

CI, confidence interval.

### Analytical specificity.

The specificity of the Aptima ZIKV assay was challenged in the presence of interfering substances to determine the analytical specificity. Clinical sera and urine from 35 patients positive for Chlamydia trachomatis, Neisseria gonorrhoeae, hepatitis C virus (HCV), hepatitis B virus (HBV), BK virus, cytomegalovirus (CMV), or human immunodeficiency virus (HIV) nucleic acid produced expected results in the presence or absence of spiked ZIKV. In addition, 46 samples spiked with yeast, bacteria, or closely related arboviruses (dengue, Kunjin, yellow fever, and chikungunya viruses) did not affect the outcome of the Aptima ZIKA assay. Collectively, the analytical sensitivity of the Aptima ZIKV assay was 100%, with no cross-reactivity or interference seen in the 81 samples tested.

### Reproducibility.

The reproducibility within the laboratory and between laboratories was tested using two panels. The intralaboratory panel consisted of 40 positive and 45 negative samples, while the interlaboratory panel contained 18 positive and 22 negative samples. The intra- and interlaboratory reproducibility results were 100% and 97.5%, respectively. The single discrepancy was reported on a urine specimen sent to an outside laboratory.

## DISCUSSION

The ZIKV epidemic continues to be problematic in many countries. As a response, many companies have developed their own diagnostic assays for detecting ZIKV infection. However, limited clinical data are available for these new assays, which makes the decision of choosing a diagnostic assay to implement challenging.

In this study, we evaluated the performance characteristics of the Aptima ZIKV assay compared to the rRT-PCR reference method. Among the 134 specimens tested by both assays, the Aptima ZIKV assay displayed excellent diagnostic accuracy, sensitivity, and specificity, which were 94.8%, 94.7%, and 94.8%, respectively. A total of seven discrepant results were found between the two assays. Although additional testing of these specimens was not performed to elucidate false-positive or false-negative results, some of the discrepant results may be attributed to the LoD of the two assays.

A critical component of the ZIKV RNA assays is their ability to detect low levels of viral RNA at the initial and final stages of viremia or viruria. The CDC reported ZIKV LoD for the Trioplex rRT-PCR assay of 2.45 × 10^3^ GCE/ml in serum and 4.64 × 10^3^ GCE/ml in urine for either small- (0.2-ml input) or large-volume (1-ml input) extractions (http://www.cdc.gov/zika/pdfs/trioplex-real-time-rt-pcr-assay-instructions-for-use.pdf). Compared to the Trioplex rRT-PCR, we found the Aptima ZIKV assay to be >100-fold more sensitive with LoD of 11.5 GCE/ml and 17.9 GCE/ml in serum and urine, respectively. This indicates that the six Aptima ZIKV assay positive and rRT-PCR negative results may be true positives. Out of these six discrepant results, four were paired serum and urine samples from two patients, which also suggests that these patients had true ZIKV infections. In addition, convalescent-phase serum obtained from one of these patients tested positive for ZIKV IgM antibodies, thus confirming the original results of the Aptima ZIKV assay, indicating that the Aptima ZIKV assay can detect ZIKV infections that would otherwise be missed by rRT-PCR.

The enhanced analytical sensitivity of the Aptima ZIKV assay may be a result of the differences in the initial testing volume. The Aptima ZIKV assay requires 0.5 ml of serum or urine for testing, while the rRT-PCR reference method tests only a small fraction of the initial 0.2-ml sample volume. Increasing the initial sample volume for extraction, input RNA for amplification, or input cDNA for RT-PCR testing may increase the sensitivity of the rRT-PCR reference method. This has recently been demonstrated by Stone et al. (12), who showed that increasing the sample input volume can improve the sensitivities of ZIKV rRT-PCR assays by >10-fold.

The main limitation in this study was the lack of ZIKV-positive patient specimens. ZIKV viremia and viruria is transient and only reliably detected within 2 weeks of symptoms or exposure (5, 6). This narrow detection window and the low risk of ZIKV infection in our service area limited the availability of ZIKV-positive specimens. As an alternative, ZIKV-negative patient specimens were spiked with ZIKV stock to enhance the robustness of the Aptima ZIKV assay evaluation. Currently, there is a proposal to test whole blood versus serum, as the ZIKV seems to last longer in whole blood, which expands the window of detection significantly (13, 14). Thus, collecting whole blood from pregnant women with concerns about ZIKV infection could be considered in the future in order to increase testing sensitivity.

The results of this study demonstrate the utility of the Aptima ZIKV assay, but the automated testing platform demonstrates the practicality of performing this assay in a clinical setting. The Aptima ZIKA assay runs on the fully automated Panther system, which performs sample preparation, extraction, amplification, detection, and results analysis. Up to 120 samples can be loaded at a time, with the ability to continuously load samples on the instrument as samples are processed. The instrument can also be interfaced with the hospital laboratory information system (LIS) to automatically post results to patients' charts. These features allow clinical laboratories to perform high-volume testing and produce faster results with less hands-on time.

In conclusion, the Aptima ZIKV assay run on the Panther system is a rapid and high-throughput method that accurately and reliably detects ZIKV RNA in patient serum and urine specimens. The automated system and enhanced analytical sensitivity of the Aptima ZIKV assay are significant advantages over the rRT-PCR reference method.

## MATERIALS AND METHODS

### Viruses, bacteria, and fungi.

The Zika virus (Asian lineage, strain FSS13025) used for the limit of detection assay was obtained from Pei-Yong Shi's laboratory at UTMB. The Zika virus Mexico (Mex-81) and Africa (DAK) strains, and the dengue virus used for diagnostic accuracy assessment were obtained from Richard Pyle's laboratory at UTMB. Dengue, Kunjin, yellow fever, and chikungunya virus vaccine strains were provided by Scott Weaver at UTMB. All other bacterial and fungal isolates were collected from the UTMB clinical microbiology laboratory.

### Clinical and spiked specimens.

All 105 clinical samples accepted for ZIKV RNA rRT-PCR were collected within 10 days of potential ZIKV exposure or symptoms and also met two thirds of the CDC's clinical and epidemiological testing criteria, which include symptoms, pregnancy, and exposure risk (see https://www.cdc.gov/zika/symptoms/index.html). Similar criteria were applied to serum samples (*n* = 20) collected from Faculdade de Medicina de São José do Rio Preto (FAMERP), São José do Rio Preto, Brazil. Spiked specimens were prepared by adding one of the Zika virus lineages (Mex-81, Cambodia strain [FSS13025], or DAK) or dengue virus into ZIKV-negative serum and urine. Users performing the Aptima ZIKV assay were blind to the identity of all specimens.

### rRT-PCR.

The rRT-PCR reference method was performed by The Texas Department of State Health Services using the CDC Trioplex rRT-PCR method or by UTMB personnel according to the protocol previously described by Lanciotti et al. (5). At UTMB, viral RNA was extracted from serum and urine specimens using the QIAamp viral RNA mini kit (Qiagen, Hilden, Germany). rRT-PCR was performed on the CFX96 real-time PCR detection system (Bio-Rad, Hercules, CA) using cDNA prepared from extracted RNA with the iScript cDNA synthesis kit (Bio-Rad, Hercules, CA). Serum or urine spiked with ZIKV was prepared alongside patient samples and used as the positive control.

### Aptima Zika virus assay.

Serum and urine samples were tested using the Aptima ZIKV assay (Hologic, Marlborough, MA) on the automated Panther system (Hologic, Marlborough, MA) according to the manufacturer's instructions. The Aptima ZIKV assay amplifies two highly conserved sequences in the NS2 and NS4/NS5 regions by transcription-mediated amplification (TMA). The entire assay is performed in a single tube and involves three main steps: sample preparation, ZIKV RNA target amplification by TMA, and detection of the amplification products (amplicons) by a hybridization protection assay (HPA). The chemiluminescence produced by hybridized probes is measured in relative light units and reported as positive or negative. Each test is equipped with an internal control (IC) to monitor nucleic acid capture, amplification, and detection. A field application specialist from Hologic provided training for the Aptima ZIKV assay on the Panther system at the UTMB laboratory.

### Limits of detection.

ZIKV stock (Asian lineage, strain FSS13025) with 2.18 × 10^9^ GCE/ml was diluted 1:100 in pooled ZIKV-negative serum and urine, and serial diluted to reach a final concentration of 1:100,000,000. Final urine dilutions were mixed with Aptima urine transport media at the ratio of 1:1 prior to testing. Multiple replicates of the lower dilutions were performed to reliably determine the limits of detection. The 95% detection probability of the GCE/ml was determined using probit analysis (see www.medcalc.org/calc/diagnostic_test.php).

### Analytical specificity.

Interfering substances included residual serum and urine from patients testing positive for bacterial or viral nucleic acid; serum and urine spiked with 1 × 10^6^ CFU/ml of commonly isolated bacteria and fungi; and serum and urine spiked with the closely related arboviruses dengue, Kunjin, yellow fever, and chikungunya (Table 4). Each sample was split into two aliquots. One aliquot was tested directly by the Aptima ZIKV assay whereas the second aliquot was spiked with ZIKV at 50 GCE/ml prior to testing.

**TABLE 4:** Interfering substances for analytical specificity analysis

Nucleic acid	Spiked organism
Chlamydia trachomatis	Candida albicans
Neisseria gonorrhoeae	Candida glabrata
BK virus	Candida krusei
Cytomegalovirus	Cryptococcus neoformans/C. gattii
Hepatitis B virus	Enterococcus faecalis
Hepatitis C virus	Escherichia coli
HIV	Staphylococcus aureus
	Streptococcus pneumoniae
	Chikungunya virus
	Dengue 2 virus
	Kunjin virus
	Yellow fever virus

### Reproducibility.

A replicate panel (40 positive, 45 negative) was made by spiking ZIKV stock into pooled ZIKV-negative serum and urine. Replicate panel tests were performed a week apart with different operators on two separate Panther systems. A second replicate panel containing 20 serum (10 positive and 10 negative) and 20 urine (8 positive and 12 negative) samples was tested using the Aptima ZIKV assay at UTMB and in a second outside clinical laboratory.

### Ethics statement.

This study was conducted under the research protocol “Evaluation/Verification of Assay and Instrument Systems Using Human Specimens” approved by the UTMB Institutional Review Board (protocol 08-182).
